# Prevalence of caries in teeth adjacent to implant-supported prostheses and relationship with implant location and the manner of restoration placement: a retrospective cohort study

**DOI:** 10.1007/s00784-026-06785-z

**Published:** 2026-03-13

**Authors:** Pablo Lastra-Prados, Jorge Cortés-Bretón Brinkmann, Diego Gómez-Costa, Luis Sánchez-Labrador, Marta Hernández de Oliveira, Jesús Rodríguez-Molinero, Antonio Francisco López-Sánchez

**Affiliations:** 1 Doctoral Program in Health Science, Faculty of Health Sciences, Rey Juan Carlos University, Avenida de Atenas s/n Alcorcón, Madrid, 28922 Spain; 2 Department of Dental Clinical Specialties, Faculty of Dentistry, Complutense University of Madrid, Plaza Ramón y Cajal S/N, Madrid, 28040 Spain; 3 Surgical and Implant Therapies in the Oral Cavity Research Group, Complutense University of Madrid, Madrid, Spain; 4 Department of Nursery and Stomatology, Faculty of Health Science, Rey Juan Carlos University, Avenida de Atenas s/n Alcorcón, Madrid, 28922 Spain; 5 High-Performance Research, Development and Innovation Group in Dental Biomaterials of King, Rey Juan Carlos University, Alcorcón, Madrid, 28922 Spain

**Keywords:** Dental implants, Dental caries, Implant-supported rehabilitation, Adjacent teeth

## Abstract

**Objectives:**

The primary objective of this study was to analyze the overall prevalence of caries in teeth adjacent to implant-supported protheses (ISPs). Secondary objectives were to determine how different implant restoration placement options (direct to implant, intermediate abutment, or Ti-Base) and/or ISP location influence the occurrence of caries.

**Materials and methods:**

This retrospective cohort study was performed at the University Clinic of Rey Juan Carlos University (URJC, Madrid, Spain), between January 2018 and December 2023, following strict inclusion and exclusion criteria. Both single crowns and fixed partial dentures supported by implants were included. Descriptive statistics were calculated for the relevant variables. Associations between different groups (direct to implant, Ti-base, and intermediate abutment) were analyzed.

**Results:**

The study evaluated 445 implant-supported protheses in 359 patients. The overall prevalence of caries in teeth adjacent to ISPs at patient level was 8.9% (95% CI: 5.95% to 11.84%) and 8.1% (95% CI: 5.56% to 10.63%) at prothesis level. Prevalence was higher in the molar region (9.6%; 95% CI: 5.7% to 13.4%), followed by premolar sites (8.6%; 95% CI: 4.2% to 12.9%). The highest prevalence of caries was found in teeth adjacent to ISPs placed direct to implant (11.4%; 95% CI: 7.3% to 15.4%); followed by teeth adjacent to implants restored with Ti-Base (10.5%; 95% CI: 0.0% to 24.2%). Those teeth adjacent to implants restored using intermediate abutments had the lowest prevalence (3.7%; 95% CI: 1% to 6.3%). Statistically significant differences between restoration groups were observed (*p* = 0.013). Moreover, the odds of caries increased by 3.37 times in teeth adjacent to ISPs placed direct to implant compared with those restored with intermediate abutments.

**Conclusions:**

The prevalence of caries in teeth adjacent to ISPs is significantly higher in implants restored direct to implant compared with those restored with intermediate abutments.

**Clinical relevance:**

Selecting the appropriate implant-supported restoration placement option may reduce caries in teeth adjacent to an ISP. Intermediate abutments would appear to reduce the risk of caries compared with direct implant restorations.

## Introduction

The rehabilitation of totally or partially edentulous jaws by means of osseointegrated implants has proven a predictable treatment option over time, making it possible to reestablish function and aesthetics without altering any adjacent dental structures [1, 2]. A recent systematic review established survival rates of 95.4% after 5 years of follow-up and 92.8% after 10 years for implant-supported fixed partial prosthesis [3].

However, dental implant treatments are not free of mechanical and biological complications [4]. Mechanical complications include the loosening or fracture of prosthetic screws with rates ranging from 3.1 to 10.8% at 5 years and the failure of various components of the implant-supported structure, such as framework fractures or ceramic abutment, with incidences varying from 3.2 to 25.5% [5, 6].

**Fig. 1:**
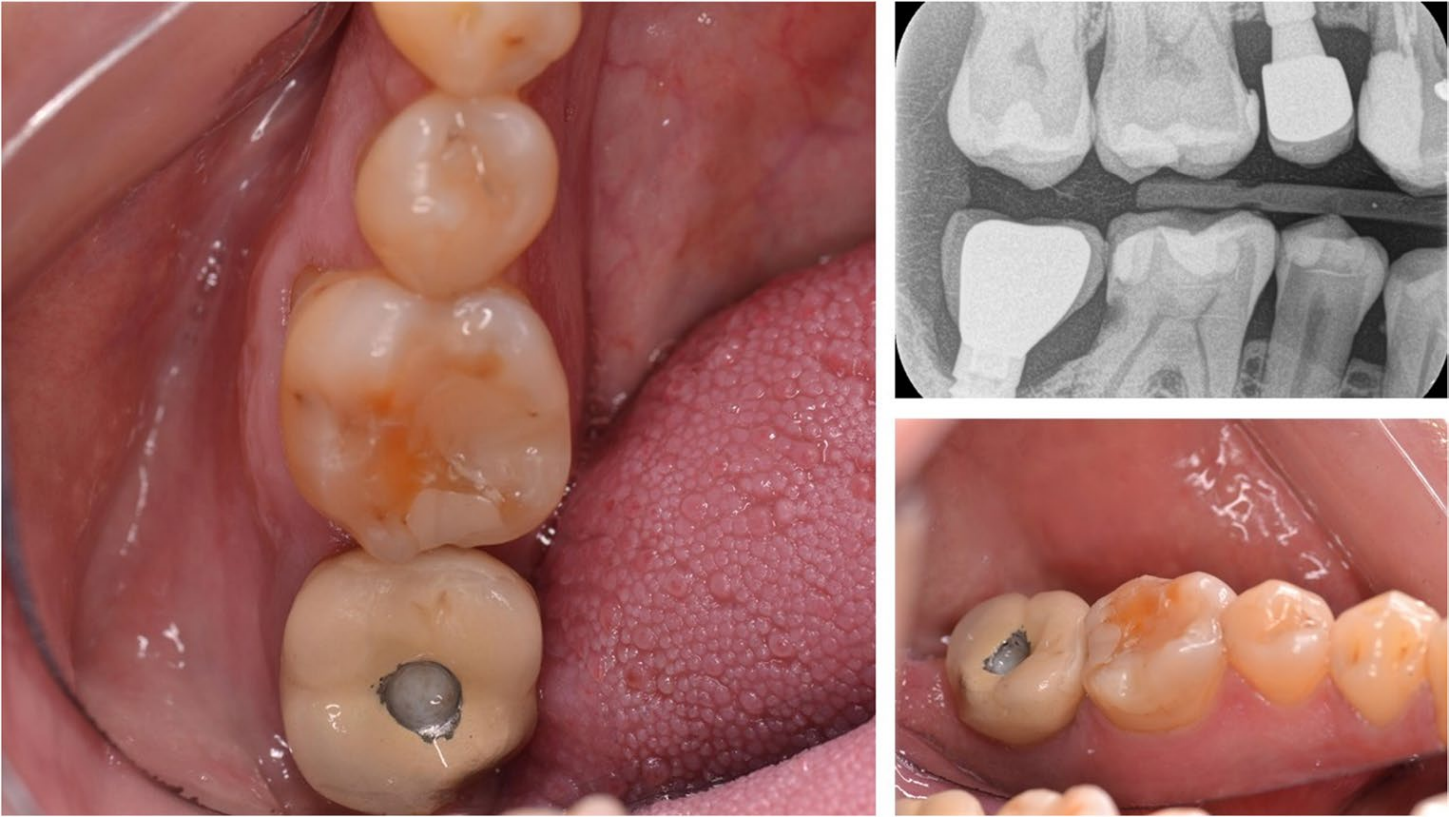
Clinical and radiological images of caries in teeth adjacent to ISPs. **A**: Occlusal vision. **B**: Bite-wing intraoral radiography showing caries in an adjacent molar. **C**: Lingual aspect

Among biological complications, infection is the most common, with mucositis affecting up to 48% of implants and peri-implantitis occurring in up to 43.9% in patients not receiving regular supportive implant therapy (SIT). However, these percentages drop to 20% and 18%, respectively, in patients who undergo regular SIT [7]. Furthermore, specific interproximal prothesis contour features may lead to food impaction. These include inadequate efficiency at the interproximal contact point with adjacent teeth (AT) or embrasure dimensions that can impede the formation and maintenance of interdental papillae and might leave open triangular spaces below the contact points [8–11]. This, together with the difficulty of maintaining hygiene in the area, can lead to the development of interproximal caries, a significant biological complication (Fig. 1). This caries often remains undetected for long periods, especially in the molar region due to poor visibility, potentially leading to extensive destruction and the eventual extraction of the AT [12, 13].

Several variables can be considered as predictors of caries in teeth adjacent to implant supported prostheses: implant location, restoration type, distance to contact point of the adjacent tooth, or simply the time elapsed since restoration placement [14–23]. It is therefore important to understand which clinical variables exert the most influence on caries formation in AT, so that preventive measures such as regular check-ups, scaling, instructing patients in oral hygiene practices, fluoride application, direct or indirect restoration with or without root canal treatment, radiographic monitoring, or even adopting different approaches to the decision-making regarding the ways in which implants are restored [14–21].

In this context, it should be noted that research directly evaluating the prevalence and risk factors of caries in teeth adjacent to ISPs is limited in scope. Many of the cited studies focus on periapical or restorative conditions rather than directly assessing the incidence of caries [16, 21]. The reported prevalence of caries in AT varies widely, ranging from 7,6% to 13,3% [22, 23].

To the authors’ knowledge, no study to date has evaluated the percentage of caries in teeth adjacent to ISPs in relation to different implant restoration options (direct to implant vs. restored with Ti-Base or intermediate abutment). The main objective of this work was to analyze the prevalence of caries in teeth adjacent to ISPs. Secondary objectives were to determine how different implant restoration options and implant locations influence the occurrence of caries in AT.

## Materials and methods

### Study design and registration

This retrospective cohort study included patients treated with ISPs at the Dental Service at the University Clinic of King Juan Carlos University (URJC, Madrid, Spain), between January 2018 and December 2023. During this period, a total of 420 patients received 520 ISPs at the study center. However, 61 patients (75 ISP) were excluded after reaching the minimum follow-up period of 1 year for various reasons. Twenty patients (32.78%; 95% CI: 21% to 44.55%) did not attend follow-up appointments after one year; in 18 patients (29.51%; 95% CI:18.06% to 40.95%), restorative treatment had to be repeated due to various mechanical problems with the prosthesis; in 14 patients (22.95%; 95% CI: 12.39% to 33.5%) the implant failed; and 9 patients (14.75%; 95% CI: 5.8% to 23.64%) were withdrawn from the study for other reasons (crowns were placed on adjacent teeth or implants were placed).

So, the final study sample comprised 359 patients with 867 implants supporting 445 fixed dental protheses, including single crowns (SCs) and fixed partial dentures (FPDs). No tooth-implant-supported FPDs were included.

Patient selection was based on pre-existing oral and radiological examinations.

All patients gave their informed consent to participate. The study was conducted following STROBE (Strengthening the Reporting of Observational Studies in Epidemiology) guidelines [24]. All procedures involving human participants fulfilled ethical standards established by institutional and national research committees in accordance with the 1964 Helsinki declaration and subsequent amendments. The study protocol was assessed and approved by the Research Ethics Committee at the URJC of Alcorcon, Madrid, Spain (Registration Code Nº 281120234232023).

Inclusion criteria were: patients aged over 18 years with implant-supported fixed restorations, who had been treated at the clinic where the study was conducted; no relevant systemic diseases (American Society of Anesthesiologists classification ASA I and ASA II); absence of caries in AT before restoration placement. Exclusion criteria were: disease or medication preventing oral surgery, pregnancy or lactation, patients who were unable to attend follow-up visits, patients rehabilitated with full-arch fixed prostheses, adjacent teeth with crowns or implants, or incomplete records.

Follow-up visits were scheduled every 6 to 12 months after prothesis delivery; subsequently, annual reviews were planned. The minimum follow-up period for patients was one year and the maximum was six years. Visits included professional maintenance (ultrasonic cleaning and supportive peri-implant and periodontal therapy tailored to individual needs), clinical and radiographic examinations for peri-implant tissue health and prothesis integrity according to ALADA (As Low As Diagnostically Acceptable) principle and ADA/FDA guidelines [25, 26]. The radiographs were taken with x-ray positioner, periapical and/or bitewing.

### Caries diagnosis

Caries diagnosis was performed by intraoral radiography as recommended in the literature [27–29]. The researcher used the ICDAS/ICCMS™ radiographic scoring system to evaluate any case of caries, as published in the ICCMS™ Guide. According to this classification, the scores are 0; RA (initial stages); RB (moderate stages); and RC (extensive stages). For the purposes of the study, any lesions observed in teeth adjacent to ISPs with a score RA3 or above were considered to constitute cases of caries, as according to the ICCMS™ Guide, these are lesions that may necessitate Tooth Preserving Operative Care (TPOC) [30, 31].

### Data collection

Intraoral radiographs were analyzed of patients who fulfilled the inclusion criteria and had been treated between January of 2018 and December 2023. The radiographs had been captured and processed with the software in general use at the University Clinic of the URJC: Carestream Dental (Carestream Dental LLC©).

One researcher (P.L.P) reviewed patients’ medical records comparing the radiographs taken at the time of prosthesis placement with those taken during follow-up. Cases of caries were identified according to ICDAS/ICCMS™.

**Fig. 2:**
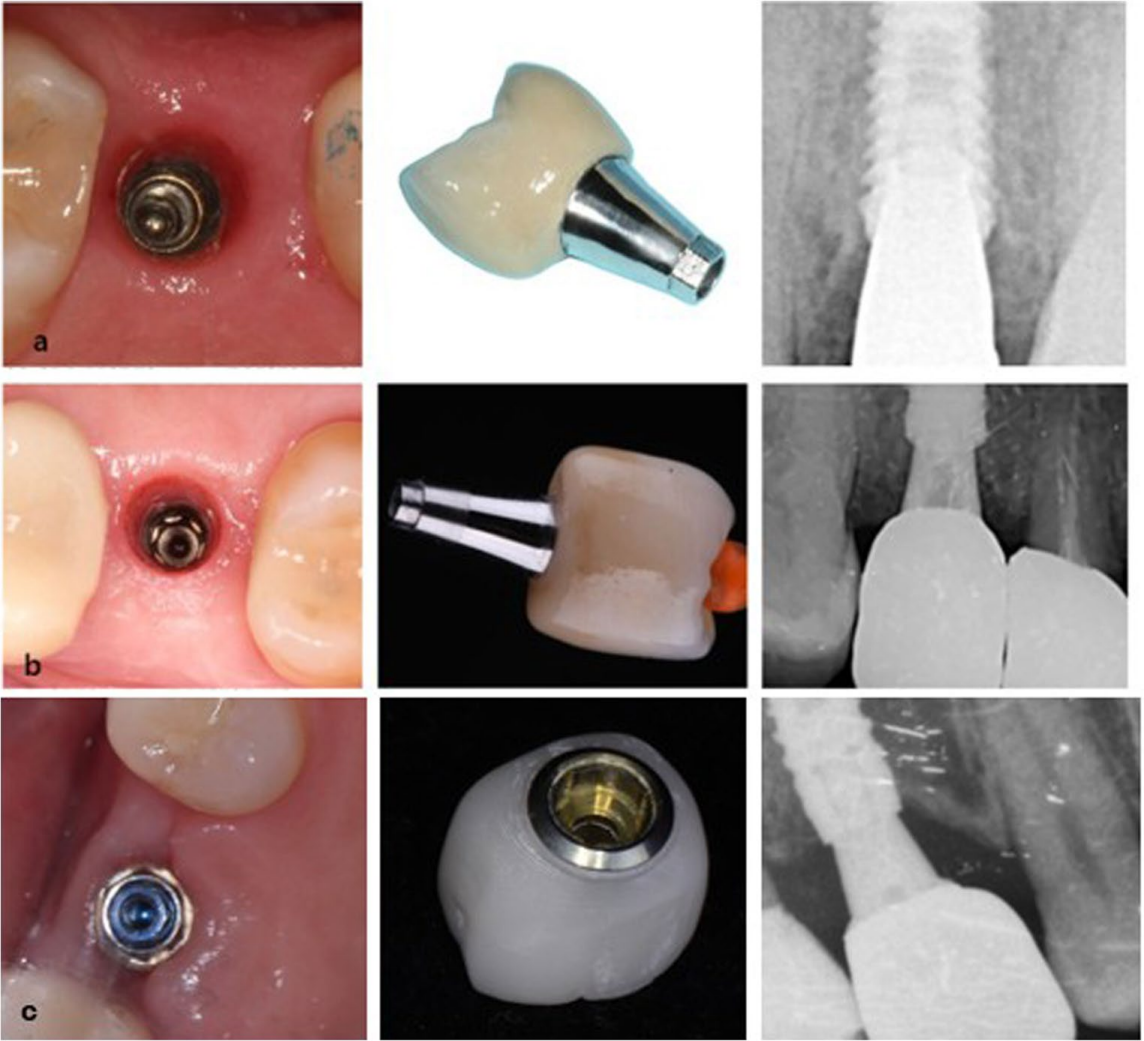
Different options for placing implant-supported restorations. From left to right: Occlusal view of the implant connection; Image of individual crown; Radiographic image: **a** Direct to implant. **b** Ti-Base. **c** Intermediate abutment

A case history was created for each subject. The following variables were collected anonymously: sex, age, date of ISP placement, type of implant restoration, ISP location (anterior, premolar, molar), type of prothesis (SC, FPD), presence of caries in AT and tooth affected (mesial, distal, both). The variable “type of restoration” was categorized as restorations placed direct to implant, restorations with prefabricated Ti-Base abutment, and restorations with an intermediate abutment (multiunit type) (Fig. 2). In addition, the time elapsed (in years) from prosthesis placement to the appearance of caries was recorded.

### Calibration

Before conducting the study, intra-examiner reproducibility (P.L.P) was established, calibrating the main variable (presence of interproximal caries) with periapical radiographs. As this was a categorical variable, the Kappa coefficient (κ) was calculated: 0.912 (95% CI: 0.81–1.00.81.00) indicating almost perfect agreement.

### Statistical analysis

Statistical analysis was conducted by an independent statistician. Data were analyzed with SPSS Statistics 29.0 software (SPSS^®^ Inc, Chicago IL, USA).

Descriptive statistics (mean, median, standard deviation, percentage distribution, confidence interval) were calculated for subject characteristics, restorations, and range of continuous data (prevalence) by implant-supported prothesis. A cumulative survival plot was constructed according to the presence of caries in teeth adjacent to dental implants using the Kaplan-Meier method. In addition, Cox regression was performed as a complement to the survival analysis to assess risk. In addition, a log-rank test was conducted to compare differences in survival between different groups. Statistical relationships were determined using the chi-square test. Statistical significance was established with a 95% confidence interval (CI) (*p* < 0.05, two-tailed).

## Results

This study analyzed data derived from 445 ISPs in 359 patients, of whom 190 were women with 223 ISPs placed and 169 were men with 222 ISPs placed.

Different brands of implants were placed, the most widely used being Klockner (30%; 95% CI: 26.9% to 33.05%), followed by AlphaBio (25%; 95% CI: 22.11% to 27.88%) and Zimvie (20%; 95% CI:17.33% to 22.66%). The least used were Bioner (10%; 95%CI: 8% to 11.99%) and Straumann (15%; 95% CI:12.62% to 17.37%).

The mean patient age was 56 years ± 11.94. Patients were monitored during a mean follow period of 3.52 years ± 1.5. The most frequent position of ISPs was molar (51.2%; 95% CI: 44.7% to 57.6%); followed by premolar (36.6%; 95% CI: 29.2% to 43.9%); and incisive/canine (11.7%; 95% CI: 2.9% to 20.4%).

Regarding the manner of implant restoration, 236 (53%; 95% CI: 46.6% to 59.3%) were restored direct to implant; 190 (42.7%; 95% CI: 35.6% to 49.7%) were restored using an intermediate abutment (multi-unit type); and only 19 (4.3%; 95% CI: 0.0% to 13.4%) were restored with Ti-Base.

**Fig. 3:**
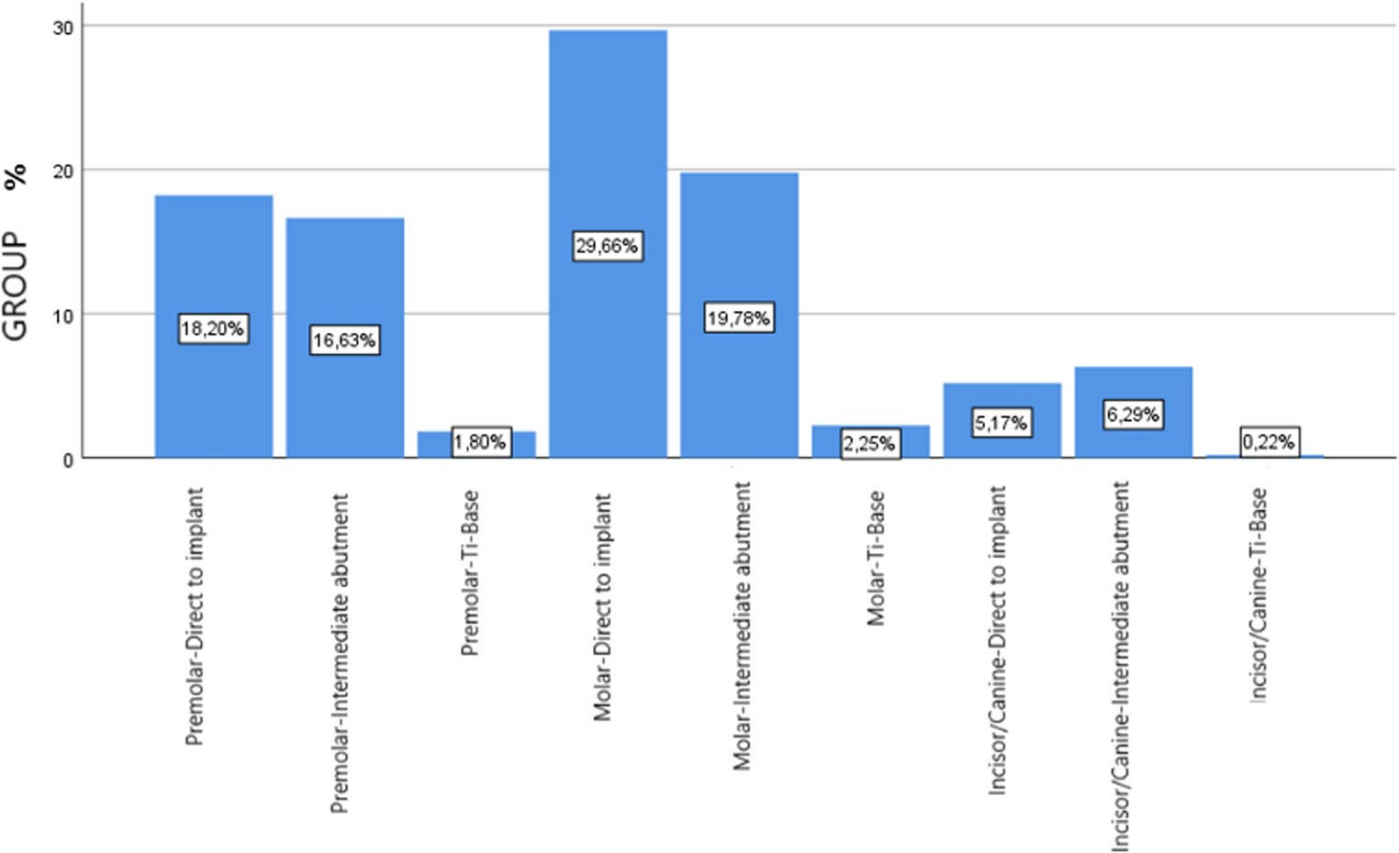
The manner of implant restoration according to position; distribution of the percentage of protheses

**Fig. 4:**
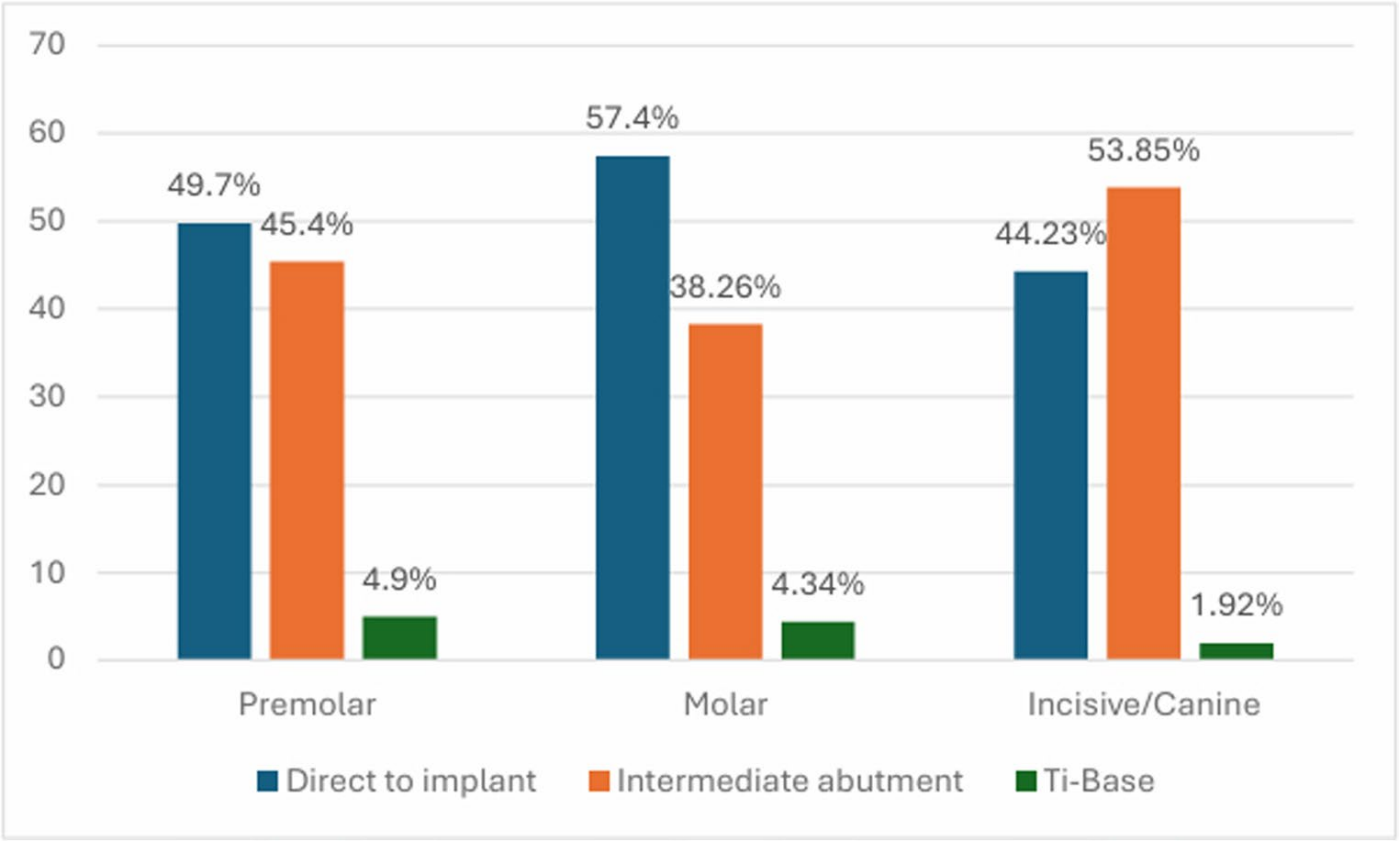
The manner of implant restoration within each group

As for implant position and manner of restoration analyzed together, for ISPs placed at molar sites, 132 (29.66%; 95% CI: 21.8% to 37.4%) were restored direct to implant; 88 (19.78%; 95% CI: 11.4% to 28.1%) were restored using an intermediate abutment; and 10 (2.25%; 95% CI: 0.0% to 11.4%) were restored with Ti-Base. For ISPs placed at premolar positions, 81 (18.2%; 95% CI:9.7% to 26.6%) were restored direct to implant; 74 (16.63%; 95% CI: 8.1% to 25.1%) were restored using an intermediate abutment; and 8 (1.8%; 95% CI: 0.0% to 11%) were restored with Ti-Base. Lastly, for ISPs placed at incisive/canine positions, 23 (5.17%; 95% CI: 0.0% to 14.2%) were restored directly to implant; 28 (6.29%; 95% CI: 0.0% to 15.2%) were restored using intermediate abutments; and 1 (0.22%; 95% CI: 0.0% to 9.4%) was restored with Ti-Base (Figs. 3 and 4).

According to type of prothesis, 207 were FPDs (46.5%; 95% CI: 41.86% to 51.13%) and 238 were single crowns (53.5%; 95% CI: 48.86% to 58.13%).

As for the FPDs, 95 were placed in molar positions (45.9%, 95% CI: 39.11% to 52.68%), 82 were placed in premolar positions (39.6%; 95% CI: 32.93% to 46.26%), and 30 were placed in incisor/canine positions (14.5%, 95% CI: 9.7% to 19.29%); while, with regard to individual crowns, 135 were placed in molar positions (56.7%, 95% CI: 50.4% to 62.99%), 81 were placed in premolar positions (34%, 95% CI: 27.98% to 40.01%), and 22 were placed in incisor/canine positions (5.9%; 95% CI: 2.9% to 8.89%).

The overall prevalence of caries in teeth adjacent to ISPs at patient level was 8.9% (95% CI: 5.95% to 11.84%) and 8.1% (95% CI: 5.56% to 10.63%) at prothesis level. The mesial teeth to the ISP were the most affected by caries (88.9%; 95% CI: 78.63% to 99.16%), followed by the distal teeth (8.33%; 95% CI: 0% to 17.35%). In 2.78% (95% CI: 0% to 8.15%) of cases, both the distal and mesial teeth were affected. These differences were statistically significant (*p* < 0.001).

The mean time of caries appearance was 4.17 years ± 1.42 after restoration placement. The mean age of patients presenting caries was 56.92 years ± 13.42.

Caries prevalence was higher in women (8.5%; 95% IC: 4.83% to 12.1%) than men (7.7%; 95% IC: 4.19% to 11.2%) although the difference between the sexes was not statistically signific ant (*p* = 0.739).

According to implant position, caries was observed in teeth adjacent to 22 (9.6%; 95% CI: 5.7% to 13.4%) ISPs placed at molar positions; in teeth adjacent to 14 ISPs placed at premolar positions (8.6%; 95% CI: 4.2% to 12.9%) but no caries occurred in teeth adjacent to ISPs placed at incisor and canine positions. The odds ratio for caries was 1.13 times higher for ISPs placed at molar positions than those placed at premolar sites (95% CI = 0.56 to 2.29). The relationship between ISP position and caries affecting AT did not exhibit statistical significance (*p* = 0.135).

The relationship between the manner of implant restoration and caries presentation showed statistical significance (*p* = 0.013). Higher prevalence was observed in teeth adjacent to implants restored direct to implant (11.4%; 95% CI: 7.3% to 15.4%), followed by teeth adjacent to implants restored using Ti-Base (10.5%; 95% CI: 0.0% to 24.2%), while the least affected by caries were teeth adjacent to ISPs restored using intermediate abutments (3.7%; 95% CI: 1% to 6.3%).

A logistic regression model was used to associate independent variables; interpretation was performed through odds transformation of the Exp(B) model. The odds ratio for caries was 3.37 times higher among implants restored direct to implants than those restored with intermediate abutments (95% CI = 1.43 to 7.93) (Table 1) and 1.09 times higher than those restored using Ti-Base (95% CI = 0.24 to 5.01); for implants restored with Ti-Base, the odds ratio was 3.07 times higher for implants restored with intermediate abutments (95% CI = 0.59 to 15.98) (Table 2).

**Table 1:** A statistically significant relationship between groups was observed. The odds of caries (Exp (B)) increased by 3.37 times when isps were restored direct to implant compared with intermediate abutment

Variables in the equation									
		B	Standard Error	Wald	df	Sig.	Exp(B)	95% I.C. to EXP(B)	
								Lower	Upper
Step 1st	Manner of restoration	1.217	0.436	7.790	1	0.005	3.377	1.437	7.939
	Constant	−4.481	0.797	31.611	1	0.000	0.011		

a. Variables specified in step 1: Manner of implant restoration.

**Table 2:** No statistically significant relationship between groups was observed although the odds of caries (Exp (B)) increased by 3.07 times when isps were restored with Ti-Base compared with intermediate abutment

Variables in the equation									
		B	Standard Error	Wald	df	Sig.	Exp(B)	95% I.C. to EXP(B)	
								Lower	Upper
Step 1st	Manner of restoration	1.124	0.841	1.785	1	0.182	3.076	0.592	15.985
	Constant	−5.511	1.889	8.506	1	0.004	0.004		

a. Variables specified in step 1: Manner of implant restoration.

**Fig. 5:**
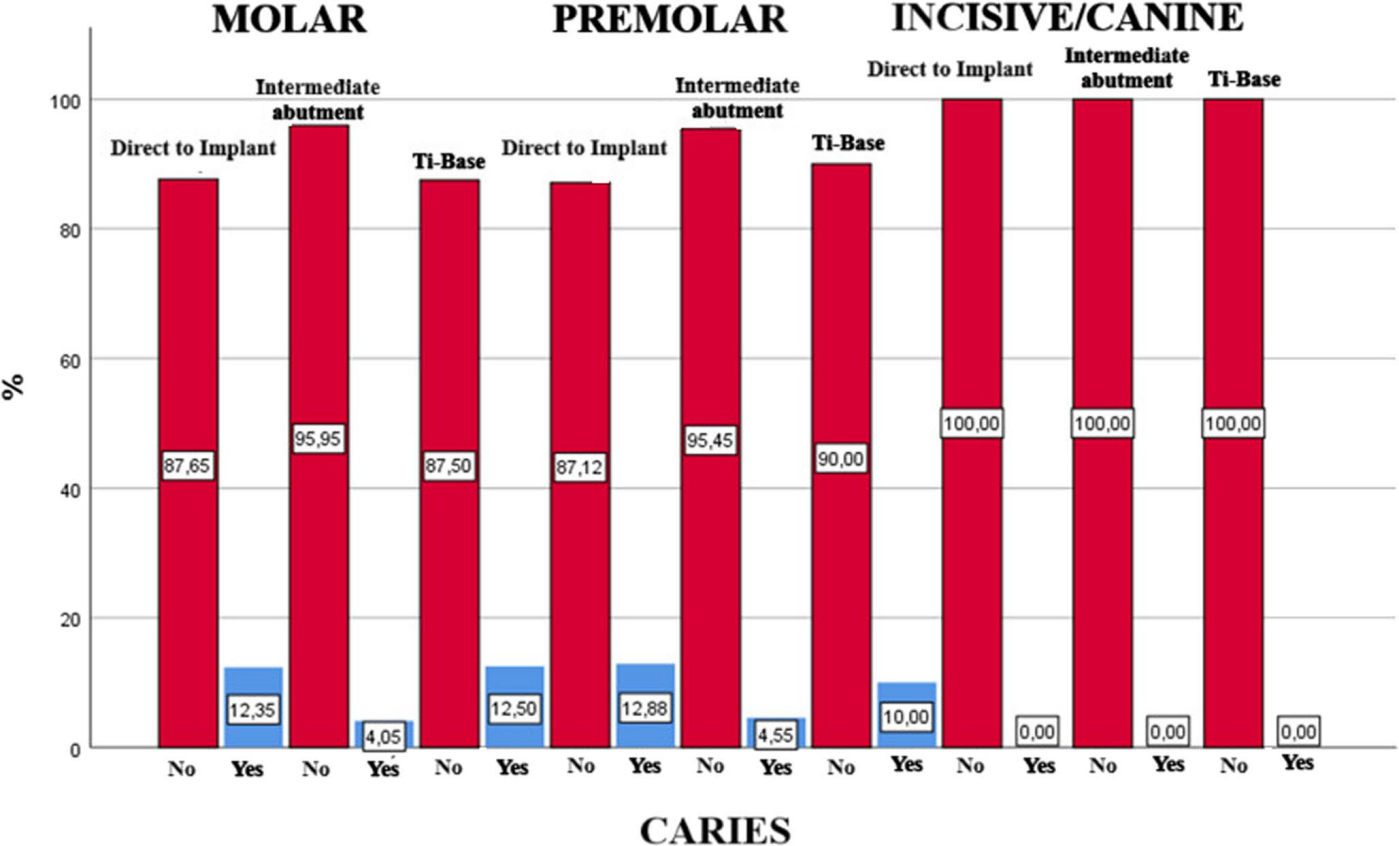
Presence of caries according to the manner of implant restoration and ISP position; distribution of the percentage of caries

Regarding ISP position and the manner of implant restoration analyzed together, the prevalence of caries was higher in teeth adjacent to ISPs at molar positions restored direct to implant (12.9%; 95% CI: 7.1% to 18.6%); followed by teeth adjacent to ISPs at premolar positions restored using Ti-Base and direct to implant (12.5%; 95% CI: 0.0% to 35.4% and 12.3%; 95% CI: 5.1% to 19.4%, respectively); the lowest prevalence of caries affecting AT was observed around ISPs at premolar positions restored using intermediate abutments (4.1%; 95% CI: 0.0% to 8.6%) and at molar positions restored using intermediate abutments (4.5%; 95% CI: 0.1% to 8.8%). However, these results were not statistically significant (*p* = 0.082). Figure 5 shows the prevalence of caries according to the manner of implant restoration and ISP position.

According to the type of prothesis, caries was found in AT in 17 FPDs (8.2%; 95% CI: 4.46% to 11.93%) and 19 single crowns (8.0%; 95% CI: 4.55% to 11.44%). The odds ratio of caries was 1.03 times higher in FPDs than in single crowns, but no statistically significant differences were found (*p* = 0.929).

The Cox regression method was used to analyze ISP position, manner of restoration, and both variables in combination. The results presented lower odds of caries in teeth adjacent to ISPs placed in anterior positions compared with posterior sites (Exp(B)=0.762; 95% CI=0.451 to 1.286) but the difference was not statistically significant (*p* = 0.308). Similarly, the use of an intermediate abutment for implant restoration presented lower odds of caries (Exp(B)=0.73; 95% CI=0.384 to 1.391) but again without statistical significance (*p* = 0.339). Lastly, it was found that a premolar position in combination with a restoration using intermediate abutment also showed a tendency towards lower odds of caries (Exp(B)=0.895; 95% CI=0.754 to 1.063), although without reaching statistical significance (*p* = 0.207).

Furthermore, the cumulative survival rate for different ISP positions was calculated by means of the Kaplan-Meier method. This found that the survival rate was higher for ISPs placed at premolar positions (5.77 years; SD = 0.72) although without statistical significance (*p* = 0.567).

Likewise, the cumulative survival rate was also calculated for the different manners of implant restoration. The results showed that the survival rate was higher for ISPs that used an intermediate abutment for restoration placement (5.87 years; SD = 0.061), although this difference did not reach statistical significance (*p* = 0.297).

**Fig. 6:**
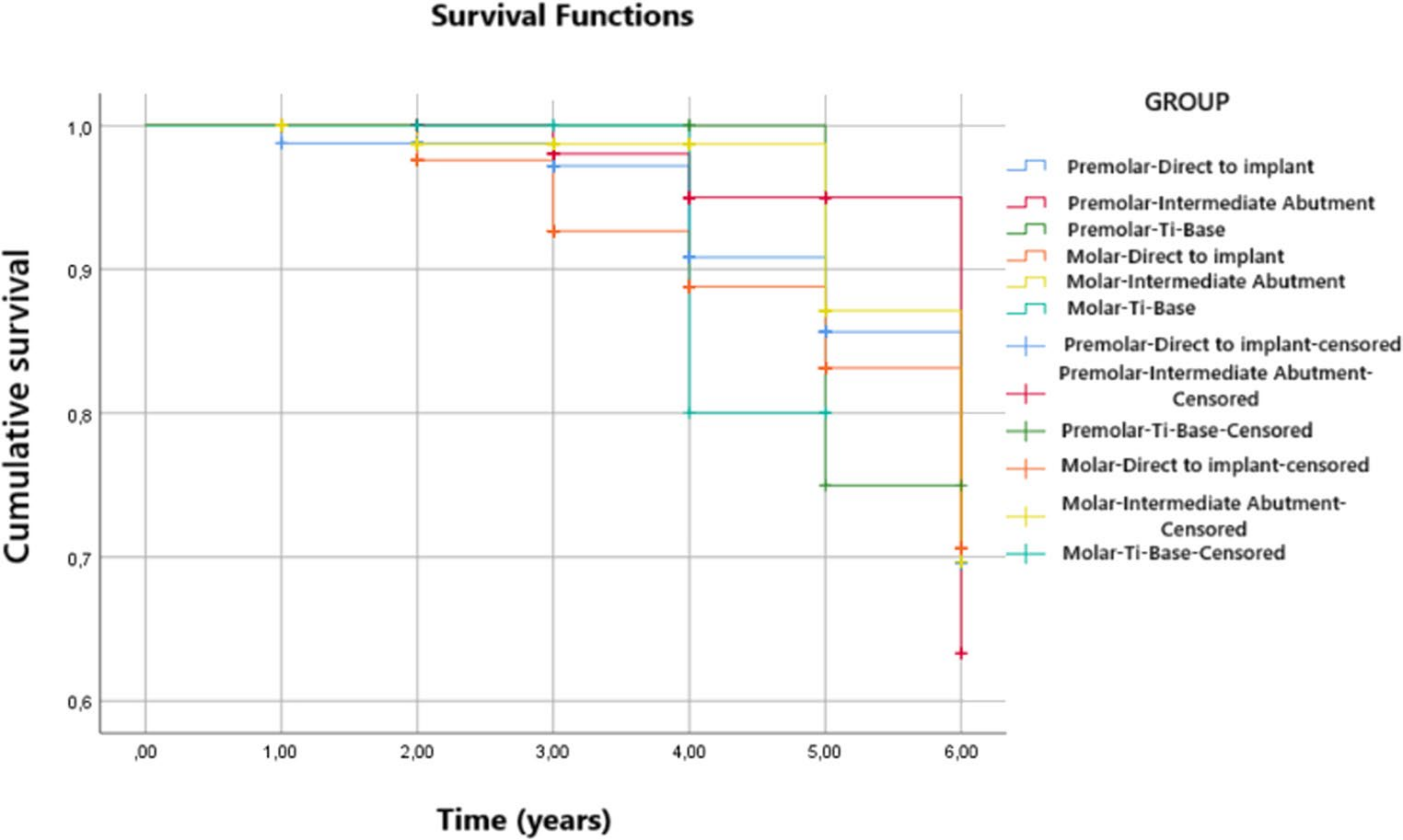
Cumulative survival rate according to ISP position and manner of implant restoration

Lastly, the Kaplan-Meier test was also applied to both variables in combination (ISP position and manner of restoration). It was found that the survival rate was higher among ISPs placed at premolar positions that used an intermediate abutment for restoration placement (5.88 years; SD = 0,102), but again without statistical significance (*p* = 0.783). (Fig. 6)

## Discussion

The aim of this retrospective study was to analyze the overall prevalence of caries in teeth adjacent to ISPs and to assess to what extent the location of the implants and different restoration placement options might influence the occurrence of caries.

According to the literature, the prevalence of caries in teeth adjacent to ISPs ranges between 7,6% and 13,3% [22, 23]. These findings are consistent with the results obtained in the present study where an overall prevalence of 8.9% at patient level and 8.1% at prothesis level was observed across 445 ISPs placed in 359 patients. Park et al. 2021 [19], in a prospective observational study, concluded that 7.5% of teeth adjacent to implants required restorative treatment, while Kim et al. 2022 [20] found that 2.9% of teeth adjacent to implants needed restorative treatment and 3.9% were extracted. Moreover, Kutkut et al. 2024 [22] reported prevalence of caries in 7.6% of teeth adjacent to ISPs in a retrospective radiographic study. It should be noted that variations in prevalence across studies may reflect differences in imaging frequency, recall intervals, and diagnostic thresholds rather than true difference in population risk.

In the present study, caries mainly affected the mesial AT (88.9%; 95% CI: 78.63% to 99.16%), followed by the distal AT (8.33%; 95% CI: 0% to 17.35%) and both sides (2.78%; 95% CI: 0% to 8.15%). These results coincide with those obtained in the study by Froum et al. 2018 [14], who documented 44 caries in the mesial teeth to the ISP and only 12 in the distal teeth out of a total of 56 affected sites. This higher caries affectation could be explained by the proximal contact loss between the ISP and the AT, which is more frequent in the mesial region [8–10]. The interproximal contact loss may lead to higher plaque and food accumulation, increasing therefore the risk of caries [12, 13].

Given the diverse locations of implant placement and the different implant restoration options in clinical use, it is essential to establish which options or implant positions are more prone to developing caries in AT.

In the present study, the mean time from restoration placement to caries appearance was 4.17 ± 1.42 years. It is to be expected that the longer the time elapsed since ISP placement, the higher the likelihood of caries development on AT. However, the existing literature shows considerable heterogeneity in the reported time lapses before caries onset. French et al. 2018 [15] reported that caries appears 3 to 5 years after implant placement. Kutkut et al. 2024 [22] obtained a mean time of 4 years (1–14 years). It should be noted that varying follow-up intervals in observational studies may influence the apparent timing of caries detection, potentially biasing estimates of caries onset. Differences in recall schedules, patients’ adherence, and diagnostic criteria may also contribute to this variability and should be considered when interpreting these findings.

In the present study, a higher overall prevalence of caries was observed in ISPs placed in the molar area (9.6%) compared with premolar (8.6%) or anterior areas (0%). This finding, although not statistically significant, would appear logical, as hygiene and maintenance procedures are more complicated in the molar sectors [32]. These results are similar to those obtained by French et al. 2018, who observed much higher affectation in posterior than anterior areas (although the study did not compare molar and premolar positions) (55:1) [15]. Smith et al. 2020 and 2021 [17, 18] and Tagger-Green et al. 2023 [23] reported caries in posterior regions but did not compare results between posterior and anterior regions.

It is also important to consider the position of the implant in relation to the distance between implant and AT, as open contact points can lead to increased plaque accumulation [15, 17, 18]. Smith et al. [17, 18], in two recent retrospective studies that assessed 300 implants radiographically, reported that caries were more frequent in those implants whose distance to AT was greater than 4 mm, suggesting that this is a critical distance.

As far as we are aware, no previous study has analyzed the possible relationship between caries in AT and the different implant restoration options commonly used in clinical practice. Kim et al. 2022 [20] observed differences between different restoration materials but did not investigate differences between the manner of restoration placement (direct to implant or abutment).

The use of intermediate abutments has been shown to reduce the microgap at the implant-abutment interface, potentially minimizing microbial leakage and preserving peri-implant tissue health [33]. Moreover, intermediate abutments enable a more favorable prosthetic contour and emergence profile, which facilitates the formation and maintenance of interdental papillae, reducing food impaction and plaque accumulation, factors that could decrease the risk of caries in AT [11–13, 33]. Additionally, intermediate abutments provide important biomechanical advantages by improving load distribution and reducing stress concentration on implant components especially under oblique masticatory forces, which are common in posterior regions [34].

In the present study, statistically significant differences in caries prevalence were observed in relation to the different placement options (*p* = 0.013). Moreover, the odds of caries in AT increased by 1.09 times in ISPs restored direct to implant compared with Ti-Base, 3.37 times in ISPs restored direct to implant compared with those restored with intermediate abutments, and 3.07 times in ISPs restored with Ti-Base compared with those restored with intermediate abutments. These results suggest a possible protective effect of the use of intermediate abutments against caries formation in AT. On the other hand, the high percentage of caries in the Ti-based restorations – much higher than initially expected – is noteworthy although the small number of cases restored in this way (only 19) point to a need for further investigation.

The results obtained in the present study are of clinical relevance when it comes to designing implant-supported restorations, especially in cases in which implants are to be placed in the molar area because of the poor access affecting hygiene measures, as mentioned above. It is strongly recommended that patients be informed about all the complications that can occur. In addition, patients should be alerted to the importance of regular check-ups, which will help prevent this complication. They should also receive adequate instruction in oral hygiene practices in order to assist prevention.

The present study suffered certain limitations, mainly due to its retrospective nature and therefore the impossibility of assessing some variables that could be of clinical relevance. One significant variable that could not be analyzed was the loss of interproximal contact points which increases the probability of caries [12–14, 17, 18] due to plaque and food impaction [35]. This loss of interproximal contact points also promotes biofilm accumulation and localized inflammation of peri-implant soft tissues [10, 12, 13], conditions which aggravate susceptibility to caries in AT. Moreover, hygiene habits, plaque index, salivary flow and smoking, prothesis contour or reasons for previous tooth loss (factors known to influence both soft-tissue health and caries development) were not available for analysis. Lastly, the limited follow-up period may have underestimated the real prevalence of caries, which could increase in longer observational studies.

While patients attended scheduled professional maintenance visits every 6 to 12 months during the first year after ISP placement and annually thereafter, including clinical and radiological evaluations and tailored peri-implant and periodontal cleaning, data on individual adherence and continuity of maintenance were not comprehensively recorded. It should therefore be noted that variability in patient compliance could represent a confounding factor.

For these reasons, further studies with longer standardized follow-up periods, ideally prospective, are recommended to analyze the different variables in a more robust way in order to provide stronger scientific evidence for the implementation of precise clinical measures and/or modification of each implant’s restoration option.

## Conclusions

The overall prevalence of caries in teeth adjacent to ISPs after a mean follow up of 4.17 years ± 1.42 was 8.9% at patient level and 8.1% at prosthesis level.

ISPs restored direct to implants have an increased risk of being associated with caries in AT (11.4%) followed by ISPs restored with Ti-Base (10.5%), the intermediate abutment option being the least at risk (3.7%). These potential risks increased when implants were placed at molar positions.

## Data Availability

All data are available in the main text or the extended data. The protocols and datasets generated and/or analyzed during the current study are available from the corresponding author on reasonable request.
