# Correction to: Prevalence of caries in teeth adjacent to implant-supported prostheses and relationship with implant location and the manner of restoration placement: a retrospective cohort study

**DOI:** 10.1007/s00784-026-06984-8

**Published:** 2026-06-24

**Authors:** Pablo Lastra-Prados, Jorge Cortés-Bretón Brinkmann, Diego Gómez-Costa, Luis Sánchez-Labrador, Marta Hernández de Oliveira, Jesús Rodríguez-Molinero, Antonio Francisco López-Sánchez

**Affiliations:** 1 Doctoral Program in Health Science, Faculty of Health Sciences, Rey Juan Carlos University, Avenida de Atenas s/n Alcorcón, Madrid, 28922 Spain; 2 Department of Dental Clinical Specialties, Faculty of Dentistry, Complutense University of Madrid, Plaza Ramón y Cajal S/N, Madrid, 28040 Spain; 3 Surgical and Implant Therapies in the Oral Cavity Research Group, Complutense University of Madrid, Madrid, Spain; 4 Department of Nursery and Stomatology, Faculty of Health Science, Rey Juan Carlos University, Avenida de Atenas s/n Alcorcón, Madrid, 28922 Spain; 5 High-Performance Research, Development and Innovation Group in Dental Biomaterials of Rey Juan Carlos University, Alcorcón, Madrid 28922 Spain

**Correction to: Clin Oral Invest**

10.1007/s00784-026-06785-z

The name for Jorge Cortés-Bretón Brinkmann were incorrectly published as:

“Jorge Cortés Bretón Cortés Bretón Brinkmann”.

The correct name should read:

Jorge Cortés-Bretón Brinkmann.

The affiliation for the authors Pablo Lastra-Prados, Jesús Rodríguez-Molinero, Antonio Francisco López-Sánchez and Diego Gómez-Costa was incorrectly listed as:

“King Juan Carlos University”.

The correct affiliation should read as follows:

“Rey Juan Carlos University”.

The authors apologize for this error. This correction does not affect the scientific conclusions of the article.

The original article has been corrected.

